# Survival of *Mycobacterium bovis* BCG oral vaccine during transit through a dynamic *in vitro* model simulating the upper gastrointestinal tract of badgers

**DOI:** 10.1371/journal.pone.0214859

**Published:** 2019-04-19

**Authors:** Gareth A. Williams, Marjorie E. Koenen, Robert Havenaar, Paul Wheeler, Sonya Gowtage, Sandrine Lesellier, Mark A. Chambers

**Affiliations:** 1 Department of Bacteriology, Animal and Plant Health Agency, New Haw, Addlestone, United Kingdom; 2 Earth, Environmental and Life Sciences, Netherlands Organization for Applied Scientific Research (TNO), Zeist, The Netherlands; 3 Department of Pathology and Infectious Diseases, School of Veterinary Medicine, University of Surrey, Guildford, United Kingdom; University of Padova, Medical School, ITALY

## Abstract

In developing an oral bait BCG vaccine against tuberculosis in badgers we wanted to understand the conditions of the gastrointestinal tract and their impact on vaccine viability. Conditions mimicking stomach and small-intestine caused substantial reduction in BCG viability. We performed *in vivo* experiments using a telemetric pH monitoring system and used the data to parameterise a dynamic *in vitro* system (TIM-1) of the stomach and small intestine. Some BCG died in the stomach compartment and through the duodenum and jejunum compartments. BCG survival in the stomach was greatest when bait was absent but by the time BCG reached the jejunum, BCG viability was not significantly affected by the presence of bait. Our data suggest that from a starting quantity of 2.85 ± 0.45 x 10^8^ colony-forming units of BCG around 2 log_10_ may be killed before delivery to the intestinal lymphoid tissue. There are economic arguments for reducing the dose of BCG to vaccinate badgers orally. Our findings imply this could be achieved if we can protect BCG from the harsh environment of the stomach and duodenum. TIM-1 is a valuable, non-animal model with which to evaluate and optimise formulations to maximise BCG survival in the gastrointestinal tract.

## Introduction

European badgers (*Meles meles*) are hosts of *Mycobacterium bovis* and represent self-sustaining wildlife reservoirs of potential tuberculosis transmission to farmed cattle and other animal species [1]. Tuberculosis (TB) infection in livestock has a significant economic impact on farming and UK taxpayers. Since badgers are protected animals under UK law, vaccination is one possible approach to managing the disease in wild badger populations and forms part of the TB eradication strategy for England [2] and Wales [3].

Parenterally delivered Bacillus Calmette-Guérin (BCG) vaccine reduces the severity and progression of experimentally induced *M*. *bovis* infection in captive badgers and also exerts a protective effect against the disease in wild badgers [4–7]. The Animal and Plant Health Agency (APHA) holds the UK Limited Marketing Authorisation for BadgerBCG, a vaccine delivered by intramuscular injection to badgers to reduce lesions caused by *M*. *bovis* infection. A more practical and cost-effective strategy for vaccination of wild animals is likely to be self-administered oral BCG vaccine in a bait deployed in the field. Oral BCG vaccination has been shown to provide protection in a number of wildlife species including badgers [8–14]. For oral uptake by badgers, the vaccine will be prepared in a food bait matrix with high palatability for badgers [15, 16].

The precise site(s) and detailed mechanism(s) of BCG uptake *in vivo* following ingestion are unclear. BCG can be phagocytosed by Peyer’s patch microfold-cells (M-cells) of the small intestine before transmission to macrophages present in underlying epithelial tissue [17]. It is therefore reasonable to assume that the badger small intestine may well serve as an important site of BCG uptake from the lumen of the small intestine during transit of the oral vaccine. Ingested BCG must be capable of surviving the physicochemical stresses of gastric and intestinal digestive conditions in the gut such that the number of live bacteria arriving at M-cells remains high enough to elicit protective immunity [18, 19]. The fact that very few BCG cells were recovered from the faeces of badgers given high doses of live BCG orally [20] suggests that the death of orally administered BCG in the gut could be limiting maximal efficacy of the vaccine. This is supported by the observation that BCG Moreau was reduced in survival by 40% and by 65% following two hour incubations in artificial gastric or duodenal solutions, respectively [21] and by our similar findings for BCG Danish in simulated gastric and intestinal fluids (this paper).

We sought to gain a deeper understanding of the specific conditions of the badger gastrointestinal tract (GIT), the impact this environment has on BCG viability, and whether viability was influenced by inclusion of the vaccine in a candidate bait delivery system. The Bravo pH Monitoring System (Medtronic Ltd) was developed originally for wireless sensing of gastric pH in humans [22] but has since been used to measure gastric pH and residence time in dogs [23, 24]. We conducted remote-sensing experiments using the Bravo System to generate data on the pH and retention times in the different compartments of the badger GIT.

As studying BCG survival *in vivo* raises both practical and ethical concerns, we instead evaluated the survival of BCG in the context of different bait formulations using the TNO TIM-1 gastrointestinal model. The TIM-1 system simulates in high degree the successive dynamic processes in the stomach and small intestine [25] and has been used previously to determine bacterial survival during gastrointestinal transit simulating human GIT conditions [26–28] and using validated (fed-state) canine digestion parameters [29]. Mixing of contents within each compartment is a key feature of the system and is achieved by alternating the pressure on flexible walls. All contractile movements and the resulting mixing and pressure profiles are accurately controlled and synchronised. To more accurately model badger digestion we refined the canine model parameters to incorporate the *in vivo* pH and gastrointestinal transit time data obtained from captive badgers using the Bravo system. We report the use of the TIM-1 system to compare the survival rate of BCG bacteria without and with different baits after passage through the stomach and the proximal and middle parts of the small intestine; the duodenum and jejunum.

## Materials and methods

All animal work was reviewed and approved by the Animal Welfare and Ethical Review Board of the Animal and Plant Health Agency and was conducted under Home Office A(SP)A licence PPL 70/5965.

### Test products

The test product was BCG Danish 1331 strain suspended in 1.5% (w/v) monosodium glutamate (MSG) solution (supplied by the Staten Serum Institute, Copenhagen, Denmark). This product was tested without bait or with three different bait formulations: PT (Pest-Tech bait [15]); HPO-PT (Hardened Peanut Oil-Pest-Tech bait); CB-PT (Cocoa Butter-Pest-Tech bait). HPO (CAS number 68425-36-5) was sourced from Sigma-Aldrich (product code 93967) and CB (CAS number 8002-31-1) was sourced from Parchem, New Rochelle, NY.

### Simulated gastric and intestinal fluids

The composition of simulated gastric and intestinal fluids are shown in S1 and S2 Tables, respectively and were derived following consultation with Dr Alex German (expert in canine gastro-physiology, University of Liverpool) and Prof Yvonne Perrie (expert in pharmacology, University of Strathclyde). Unpublished measurements obtained by us from badgers *post mortem* indicated the typical volume of a badger stomach is approximately 500 ml. Therefore, this volume of solution was used in the *in vitro* tests. To examine the specific action of pepsin on BCG, a basic gastric fluid was made from a solution of pepsin (0.32%, w/v) and the pH adjusted to 2.5 using hydrochloric acid. To examine the effect of bile on BCG, ox bile (Sigma-Aldrich, product code 70168) was used as a 10% solution to represent the physiological concentration of fresh bile in the proximal small intestine. As the pH of the ox bile solution was 7.5, a control solution of KH_2_PO_4_ was included in this experiment to match the pH, as the pH of the MSG solution containing BCG is 6.8.

### Bravo pH monitoring system

The Bravo pH Monitoring System (Medtronic, Minneapolis, MN) consists of a capsule (26×6×6.3 mm), which is placed in the GIT using a catheter delivery system from where it measures the pH of its environment; transmitting the data to a remote recording device [30]. Proof of principle was first demonstrated with the system by placing the capsule in the stomach of a dead lamb (approx. 30 kg) and confirmed to be transmitting to the recording device. The lamb (double-bagged) was taken to the badger holding facility and placed in various locations around the compound, including within the wooden setts used by the badgers (with badgers excluded). An optimal location for the recording device was identified that allowed the transmitted pH data to be recorded at all positions within an enclosed area of the facility. Badgers have previously been confined to this enclosed area for several weeks without problem, indicating the system could be employed to record data directly from the GIT of live badgers in the facility.

Following Home Office approval (licence, PPL 70/5965) the work progressed to studies in captive badgers. Before use, each capsule was calibrated against a Mettler Toledo benchtop pH meter (Fisher Scientific) in standardised buffer solutions between pH 2 and pH 10, achieving a Pearson correlation coefficient of greater than 0.98. Capsules were placed in badgers whilst under anaesthesia, deep in the oesophagus by intubation (insertion depth = 30-35cm) using the sterile applicator supplied with the capsule. After a capsule was positioned, each badger was returned to a 42 m^2^ enclosure for recovery and was free to feed and interact normally with other captive badgers. The receiver was hung in the middle of the room out of reach of the badgers within reception range (at least 5 m) from the capsule and data collected for up to 48 h. In all cases, the capsule was recovered from faeces deposited in the enclosure. Data were recovered from the receiver using a PC and visualised using the manufacturer’s software.

### Test system: Dynamic gastrointestinal system (TIM-1)

Experiments were performed in the TNO dynamic, multi-compartmental system of the stomach and small intestine (TIM-1) as schematically presented in S1 Fig. This system simulates very closely the successive dynamic conditions in the stomach and small-intestinal tract, such as body temperature, the pH curve, concentrations of electrolytes, and the activity of enzymes in the stomach and small intestine, the concentrations of bile salts in the different parts of the gut, and the kinetics of transit of the chyme through the stomach and small intestine, and the absorption of low molecular weight molecules and water. For the experiments described in this paper, the sampling bottle was placed under peristaltic valve F instead of H (S1 Fig), in order to sample bacteria entering the ileum.

**Fig 1:**
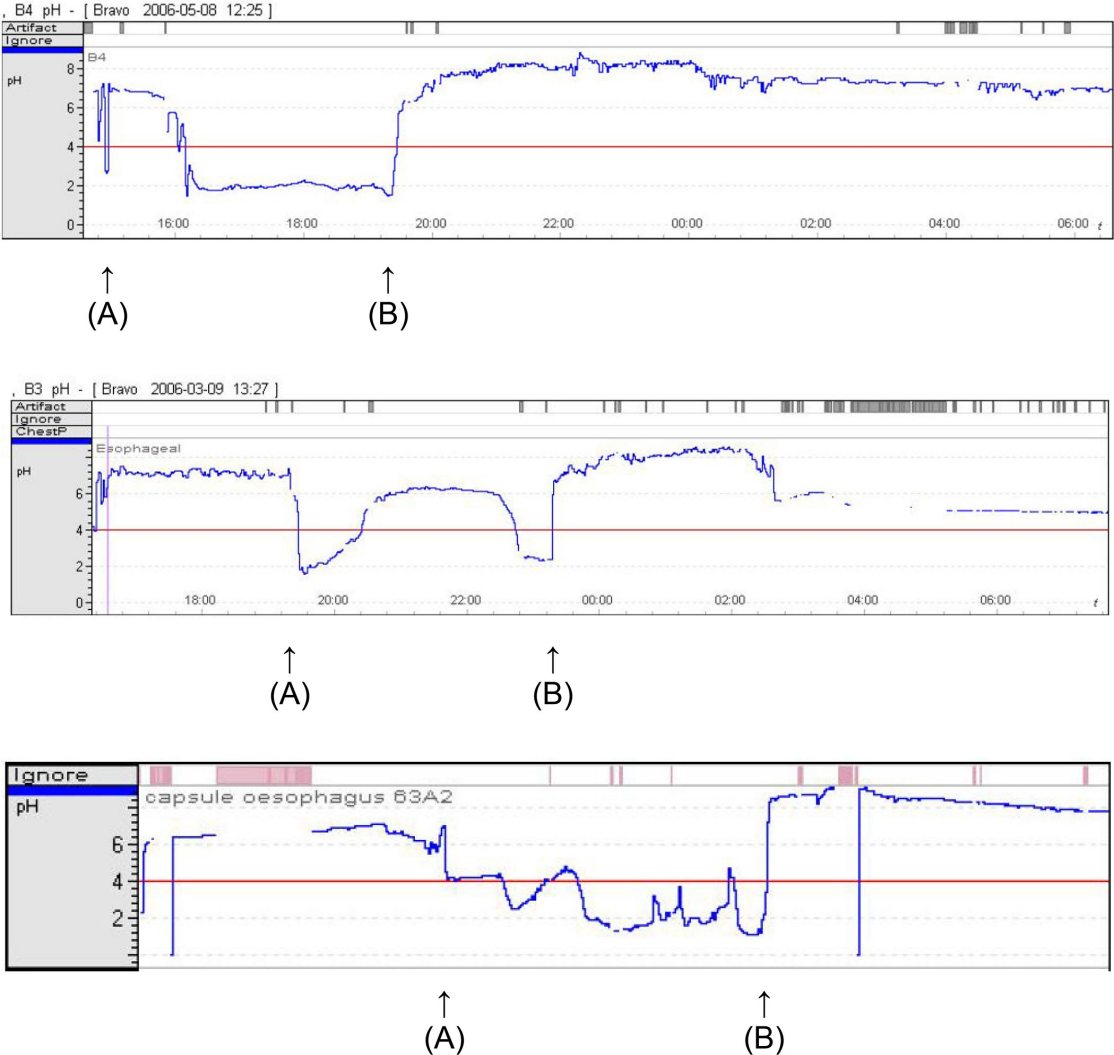
Traces of pH versus time obtained from three different live badgers using the Medtronic Bravo pH Monitoring System. (A) Capsule moves into stomach, pH declines. (B) Capsule moves from stomach into small bowel, pH rises sharply. Capsule in stomach between points A and B.

The experiments in TIM-1 were performed under the average physiological conditions of the stomach and small intestine as described for dogs in the fed state (light meal) [29] with adaptations according to data from three badgers which were obtained by us using the Medtronic Bravo pH Monitoring System. Data for the dynamics of gastric emptying and intestinal transit times, and the gastric and the intestinal pH values were used to set the parameters for TIM-1 (Fig 1).

The gastric contents passed the pyloric valve into the duodenal compartment according to the computer controlled gastric emptying, an exponential equation defining a t½ for the half time of delivery and ß for the shape of the curve [31]. In this study t½ = 70 min and ß = 1 were used. As such, 99% of the stomach contents had passed into the duodenal compartment by 8 hours. In the duodenal compartment the gastric content was neutralised to pH 6.3 ± 0.2, and bile and pancreatin were secreted. The content of the duodenum (passage time ± 10 min) was gradually delivered into the jejunum (pH 7.2 ± 0.2) compartment. After a jejunal passage time of approximately 90 min, the contents was emptied in a beaker on ice containing dilution buffer (physiological salt with 0.05% Tween) in order to immediately dilute the enzymes and bile. In each compartment the physiological concentrations of bile salts, pancreatic enzymes and electrolytes were simulated in combination with the average physiological passage of the chyme through the small intestine. The digested and soluble (low-molecular weight) compounds were removed continuously from the jejunum compartment of the model via a special dialysis membrane system.

For each experiment the secretion products (e.g. salivary and gastric juice with enzymes, electrolytes, dialysis liquids, bile and pancreatic juice) were freshly prepared, the pH electrodes calibrated and the dialysis membrane (hollow fibre units) replaced.

### Cleaning and decontamination of the TIM-1 system

The cleaning procedure of the TIM-1 system was adapted to the need for plating bacteria. As sterilising the system is not possible, the system was cleaned after each run, first with water and thereafter filled with a soap solutions (0.1 M NaOH in 2% RBS solution) and left in the cleaning solution overnight.

The day before an experimental run, the glassware of the system was clicked off the machine and put in the sink. The compartments were cleaned with water and after that put in a bleach solution for at least 30 minutes. After this the units were rinsed with water, then with 0.5 M HCl, then with water and finally with 70% ethanol. The units were dried and kept dry and empty until use. Just before use all electrodes, sensors and caps were rinsed with 70% ethanol and dried.

### Set up of the experiments

Two separate studies were performed. In Study 1, four replicate experiments were performed over two days and included the evaluation of BCG, BCG plus HPO-PT bait and BCG plus CB-PT bait. In Study 2, a further four replicate experiments were run over four days and included the evaluation of BCG, BCG plus HPO-PT bait and BCG plus PT bait. In Studies 1 and 2, BCG was recovered in effluent from the jejunum. In Study 2, BCG was also recovered in effluent directly from the stomach via the pyloric sphincter (S1B Fig).

Baits were taken out of the refrigerator the night before the TIM experiments to warm to room temperature (approx. 21°C). The baits were cut into four parts, then squeezed through a strong garlic crusher in order to simulate the chewing by badgers. The artificial saliva and water were put in a water bath until the temperature was between 42 and 45°C. The ‘chewed’ bait was added, the mixture stirred and then introduced into the stomach of the TIM-1 system via a funnel. When the temperature in the stomach decreased to 37°C, 400 μl of BCG suspension was introduced into the stomach compartment. For experiments without bait matrix, it was replaced with 30 g of citrate buffer (0.01 M concentration in the stomach content). Each of the three vaccine preparations was tested in at least duplicate. The total intake in the gastric compartment of the system was 300 g in all runs.

### Sampling and sample treatment

Before the TIM experiments, per test day two samples of the test product were taken and analysed for the number of viable BCG ml^-1^, in order to determine the CFU’s in the intake for each TIM-1 run retrospectively. During the TIM run, the gastric effluent (Study 2) and the jejunum effluent (Studies 1 and 2) were collected in one hour aliquots into sampling bottles with 100 ml of dilution buffer. In this way the sample was diluted in order to minimize the effect of bile and pH in the sampling bottle on survival of BCG after passage through the TIM system. In Study 1 the dilution buffer was phosphate buffered saline with 0.05% Tween on ice. In Study 2 the dilution buffer was Sauton medium with 0.05% Tween. Samples were collected at room temperature to limit the opportunities for fat to congeal in the sample. Every 60 min the collected volume was measured and sampled. The residues in the TIM system, the lumen present in the gastric compartment and from the combined duodenal and jejunum compartments after ending the TIM experiment, were collected for analysis.

### Microbiological analyses

Samples were diluted in Sauton-Tween and plated in duplicate on Modified 7H11 agar plates in order to determine the number of CFU of BCG. The plates were closed with tape around the edge and incubated in sealed bags for 28 days at 37°C before counting the number of colonies on each plate. For TIM-1 experiments, the survival rate of the BCG bacteria after passage through the stomach, duodenum and jejunum was determined in at least duplicate for each run, making adjustment for the volume of the effluent in each case.

### Data calculation and statistics

After incubations of the plates, the BCG colonies were identified morphologically and counted in duplicate. Differences between mean counts were evaluated by Student t-test or 2-way ANOVA using GraphPad Prism version 7.03 for Windows, GraphPad Software, La Jolla California USA, www.graphpad.com.

## Results

**Fig 2:**
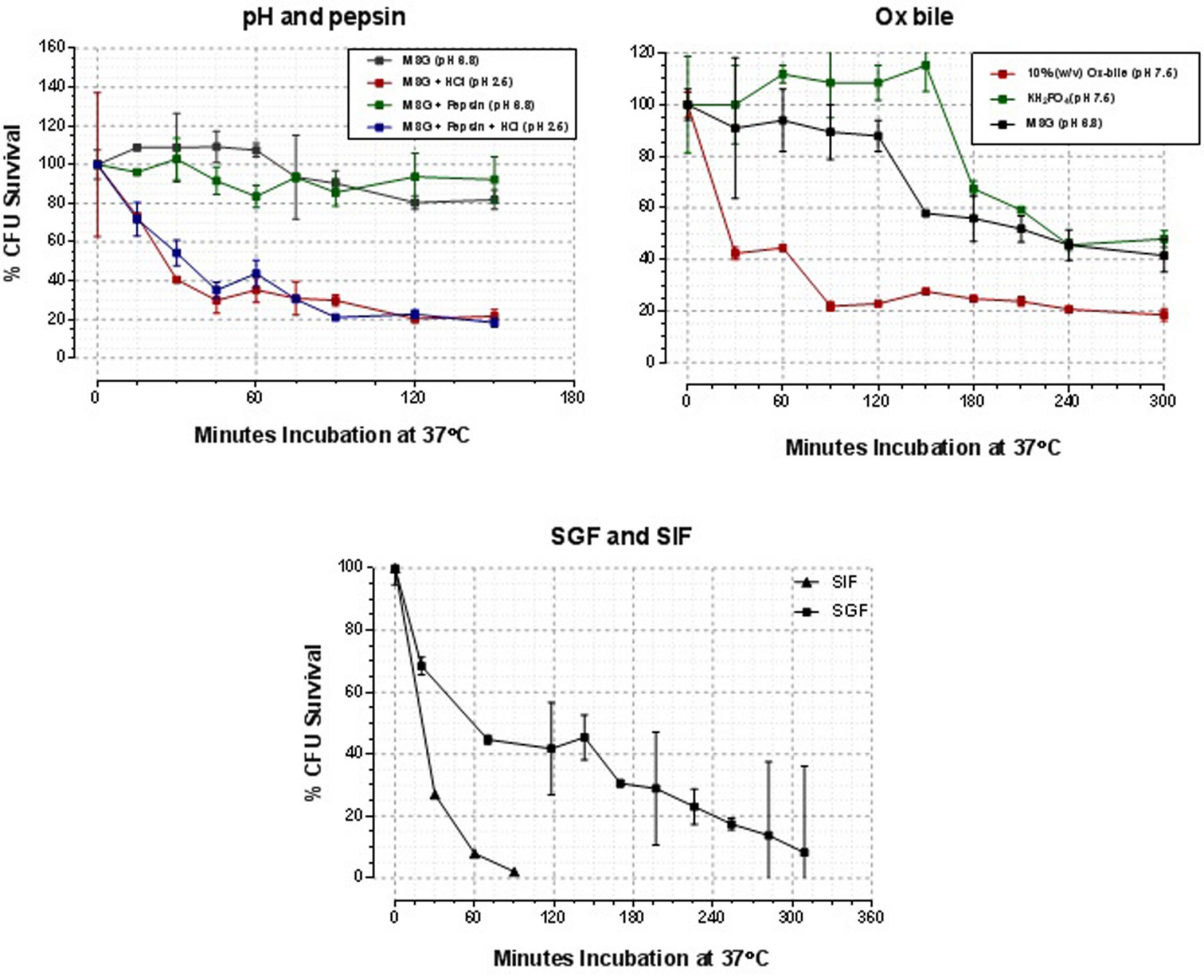
The impact of pH, pepsin, Ox bile, SGF and SIF on the viability of BCG Danish. Data are expressed as percentage survival of BCG from the original starting inoculum. Data are expressed as the mean values derived from quadruplicate samples and bars indicate the standard error around the mean. The data used to generate this figure are provided in S1 Dataset.

Initially, we performed simple *in vitro* experiments using different fluids designed to mimic the low pH and enzymatic activity of the stomach and duodenum. We wanted to ascertain whether these conditions would cause a significant reduction in BCG viability and consequently could have a detrimental effect on the protective efficacy of an oral BCG vaccine. Fig 2 shows the results of these experiments. Exposure of BCG Danish to pH 2.5 caused significant reduction in BCG viability within 15 min (p < 0.05, t test), reaching a maximum after about 120 min. This was not increased by the inclusion of pepsin. A similar reduction in viability was seen on incubation of BCG with ox bile, where a reduction in viability of 80% occurred after 100 min of incubation, despite BCG originally being derived from virulent *M*. *bovis* using medium containing ox bile [32]. Reductions in BCG viability were even greater (>90%) when more complex solutions designed to simulate gastric or intestinal fluids were used.

In order to gain more accurate information for the conditions which BCG vaccine may face in the GIT of badgers, we performed *in vivo* remote-sensing experiments on three different badgers using a Bravo pH Monitoring System (Medtronic Ltd); a telemetric capsular system that transmits sensed pH readings in real-time. Fig 1 shows the traces obtained with the Bravo device. The capsule was placed in the oesophagus of badgers under anaesthesia. After recovery the badger was released into its enclosure and observed using CCTV cameras. The demarcations between entry into and exit from the stomach were easy to identify on the basis of rapid reduction and increase in pH, respectively. Readings of pH from the capsule varied between animals as might be expected. In general, pH reached a low of 2 in the stomach and a high of 8 in the duodenum. The data across the three badgers were: pH7 within the oesophagus; reduction of pH from 7 to 2 within the stomach over a mean period of 60 min (range 10 to 105); mean gastric retention time of 480 min (range 270 to 720); increase of pH from 2 to 8 over mean of 75 min (range 15 to 120) on passage from stomach into duodenum. We were unable to determine the total retention time in the intestine but it was in excess of ten hours.

**Fig 3:**
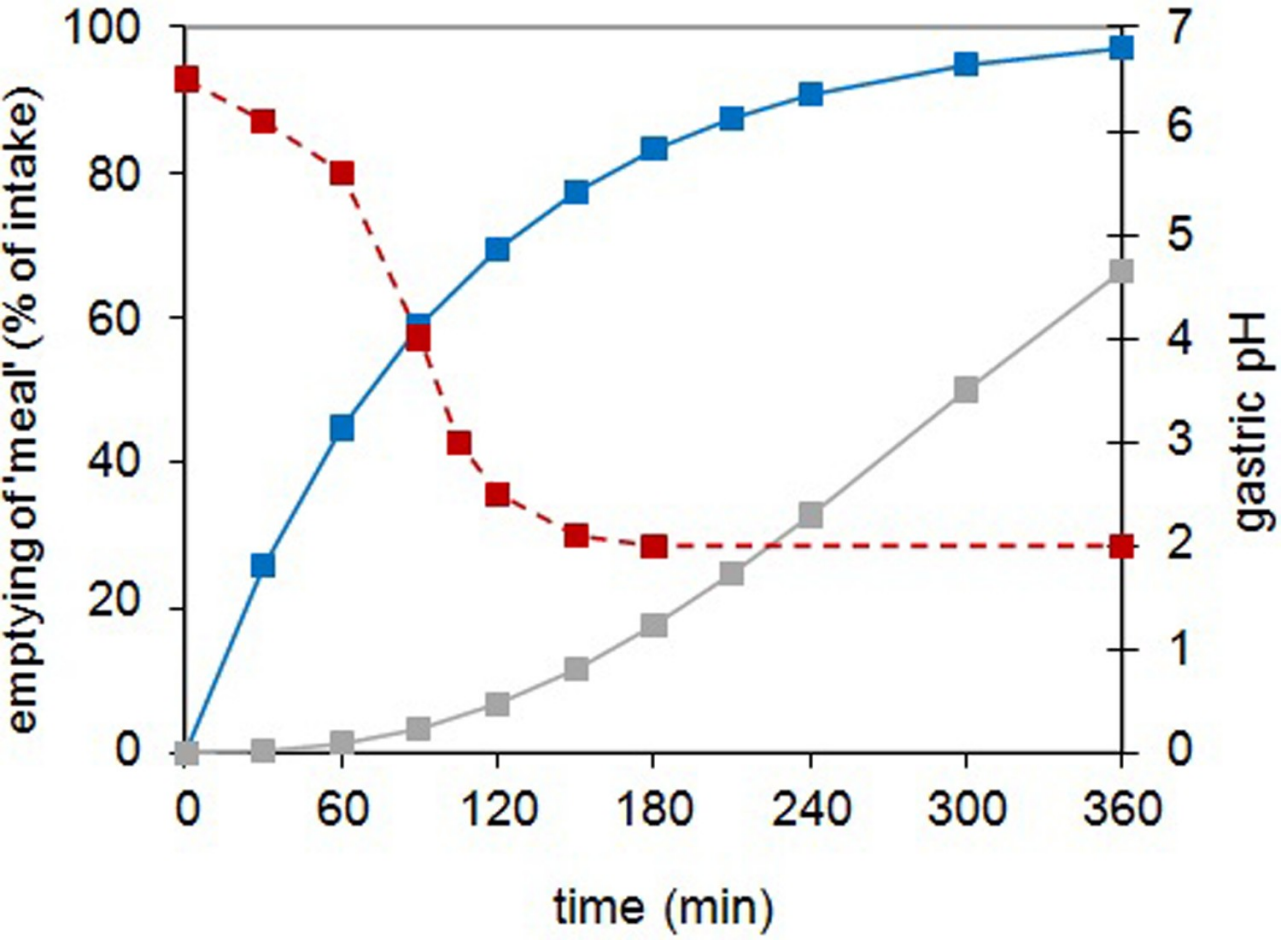
The average physiological gastric emptying curve (blue line), ileum effluent curve (grey line) and gastric pH curve (red dotted line) for badgers after the intake of a light meal as used in the TIM-1 experiments.

We have developed and evaluated a number of different bait delivery systems and found that bait, in-part comprised of peanut butter, cereal, and sugars, is palatable to badgers and compatible with long-term storage of viable BCG, especially when the vaccine is enrobed within a lipid carrier within bait [15, 16]. The physicochemical properties of the bait and vaccine carrier components of an oral vaccine may help to overcome the problem of BCG inactivation within the GIT; for example the presence of lipid in vaccine formulations may help to protect BCG against the bactericidal effects of gastric secretions and enhance its uptake through the intestinal wall [33]. We evaluated the survival of BCG in the context of different bait formulations using the TNO TIM-1 gastrointestinal system. To more accurately model badger digestion we attempted to refine the canine model parameters to incorporate the *in vivo* pH data obtained from captive badgers and to approximate the gastric retention time. In reality it proved difficult to replicate the conditions of the badger exactly since there was considerable variation between animals (Fig 1). An average gastric retention time of 480 min was modelled successfully by the formula y = a2^-t/70^, such that 99% of the gastric content was predicted to have passed into the duodenum by eight hours. The dynamics of gastric pH was harder to model and in practice it took the model three hours to reach pH2 (Fig 3); slower than observed *in vivo* (Fig 1).

A total of eight TIM-1 experiments were performed over six days on two separate occasions as part of two independent studies. The number of viable bacteria in the BCG suspension was determined for each experiment in duplicate and ranged from 4.4 x 10^8^ CFU ml^-1^ to 9.7 x 10^8^ CFU ml^-1^. The variation in CFU of BCG between the days of each study was less than between the studies, therefore we used the average number of CFU across the days to calculate the intake CFU for each study: 5.9 x 10^8^ CFU ml^-1^ (SD = 1.0 x 10^8^ CFU ml^-1^); and 8.2 x 10^8^ CFU ml^-1^ (SD = 3.6 x 10^8^ CFU ml^-1^), respectively. As 400 μl was introduced into the TIM-stomach, the average intake of BCG for Study 1 was 2.4 x 10^8^ CFU and 3.3 x 10^8^ CFU for Study 2.

**Fig 4:**
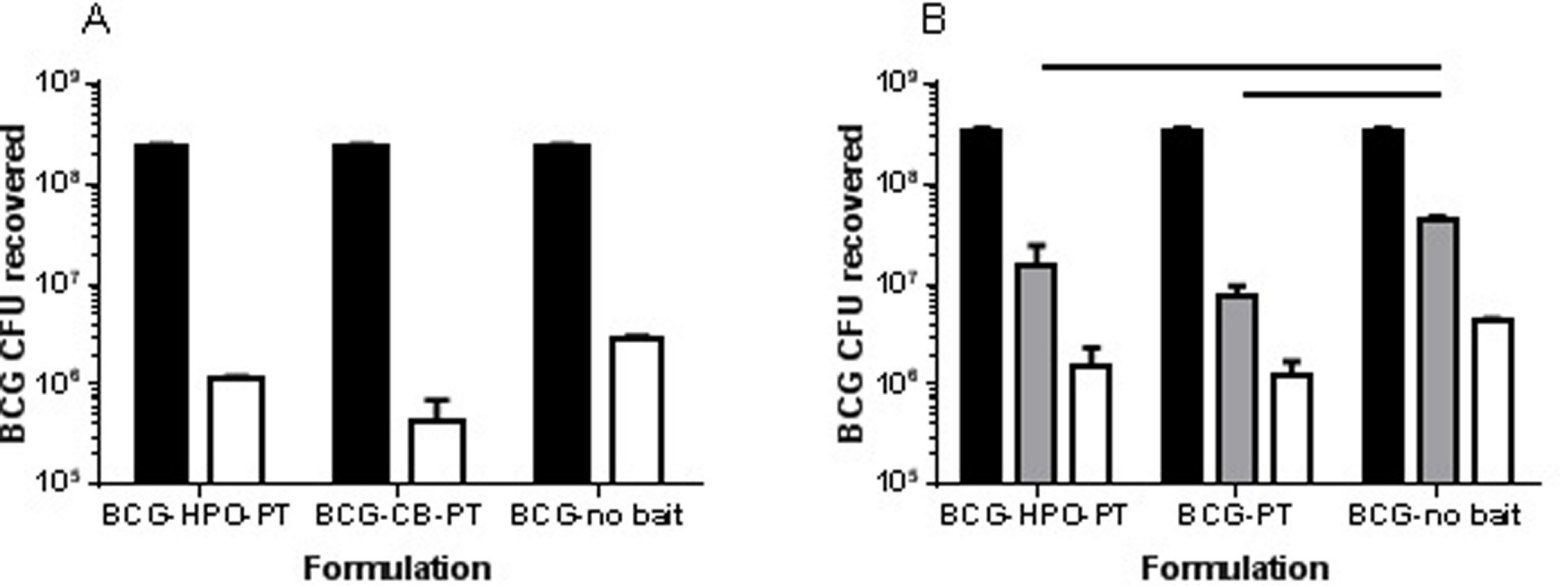
Recovery of BCG from the TIM-1 system (mean CFU plus standard error shown). BCG was recovered from three separate experiments. (A) Results from Study 1 comparing recovery from the jejunum after five hours of passage from the stomach through the duodenum to the jejunum (white bars, n = 2). (B) Results from Study 2 comparing recovery from the stomach after three hours (grey bars, n = 3), or from the jejunum after five hours of passage from the stomach through the duodenum to the jejunum (white bars, n = 3). In both cases, the black bars represent the input quantity of BCG ‘fed’ to the TIM-1 system (n = 2). BCG was introduced in three different bait formulations (HPO-PT, CB-PT, PT) or without bait. Horizontal lines indicate means that are significantly different from one another (p < 0.05). The data used to generate this figure are provided in S2 and S3 Datasets.

Table 1 presents the net survival of BCG after passage through the TIM stomach alone (following the gastric emptying for three hours) or after passage through the stomach, duodenum and jejunum compartments (following the gastric and small-intestinal passage for five hours) for all formulations tested. The log_10_ reduction in survival for BCG in HPO-PT bait (2.3) or without bait (1.9) was identical between the two independent studies, demonstrating the high reproducibility of the model. Losses to BCG survival were incurred both in the stomach compartment and subsequently through the duodenum and jejunum compartments (Table 1 and Fig 4). According to 2-way ANOVA of the data from both studies, for each formulation there was a significant reduction in BCG survival following passage through the stomach, duodenum and jejunum. The second study allowed us to determine that passage through the stomach made the greatest contribution to this reduction. By 2-way ANOVA, for each formulation, significantly less BCG was recovered after passage through the stomach, but the difference between BCG recovered from the stomach and the quantity of BCG recovered from the jejunum was not significant.

**Table 1:** Overview of the CFU of BCG Danish vaccine in the intake and the amount of CFU surviving passage through the stomach, duodenum and jejunum averaged across four experiments in TIM-1 simulating badgers GI conditions.

Intake formulation	Average intake CFU	Average surviving CFU	Log_10_ reduction in survival
Study 1:		Stomach -> jejunum (5 hrs)	
BCG-HPO-PT	2.4 x 10^8^ CFU	1.2 x 10^6^ CFU	2.3
BCG-CB-PT	2.4 x 10^8^ CFU	4.3 x 10^5^ CFU	2.7
BCG-no bait	2.4 x 10^8^ CFU	2.8 x 10^6^ CFU	1.9
Study 2:		Stomach only (3 hrs):	
BCG-HPO-PT	3.3 x 10^8^ CFU	1.6 x 10^7^ CFU	1.3
BCG-PT	3.3 x 10^8^ CFU	7.6 x 10^6^ CFU	1.6
BCG-no bait	3.3 x 10^8^ CFU	4.3 x 10^7^ CFU	0.9
Study 2:		Stomach -> jejunum (5 hrs):	
BCG-HPO-PT	3.3 x 10^8^ CFU	1.5 x 10^6^ CFU	2.3
BCG-PT	3.3 x 10^8^ CFU	1.2 x 10^6^ CFU	2.4
BCG-no bait	3.3 x 10^8^ CFU	4.5 x 10^6^ CFU	1.9

**Fig 5:**
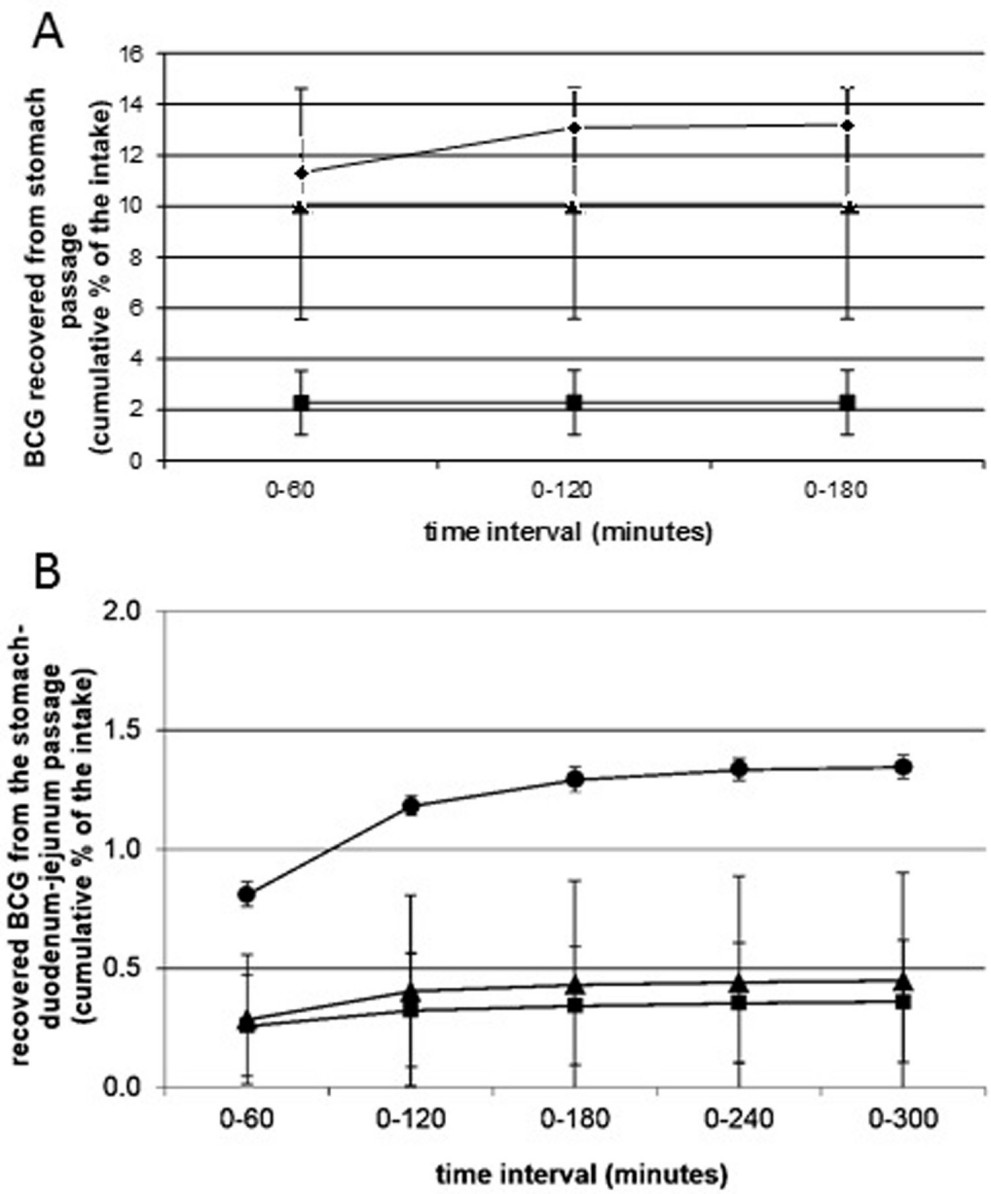
Mean cumulative number of BCG in the stomach effluent (A) and jejunum effluent (B) as percentage of the intake for Study 2, n = 3, mean ± stdev for BCG-HPO-PT (triangles), n = 3, mean ± stdev for BCG-PT (squares) and n = 2, mean ± range for BCG without bait (circles).

**Fig 6:**
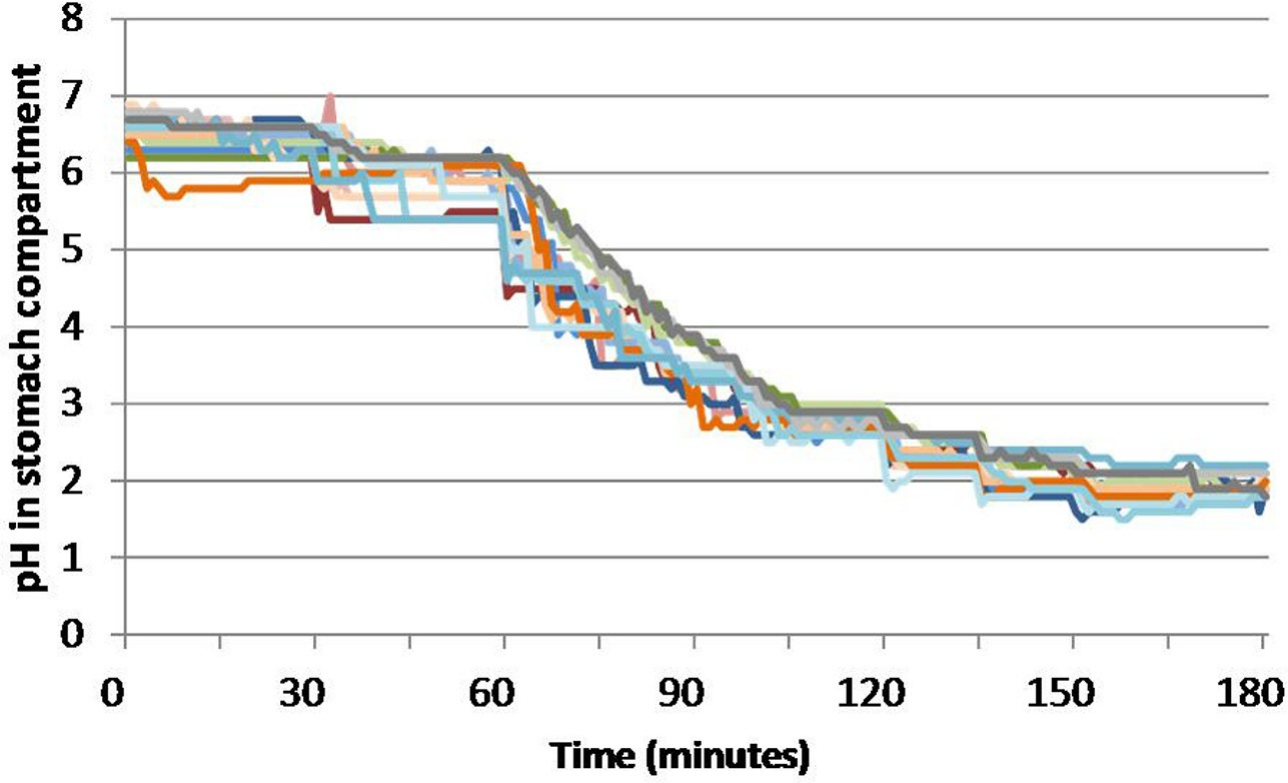
pH in the stomach compartment of the TIM-1 system during all runs. BCG-HPO-PT (six shades of red and orange), BCG-PT (six shades of blue) and BCG without bait (2 green and 3 grey shades).

As passage through the stomach made the greatest contribution to a reduction in BCG viability, we specifically assessed the survival of BCG in three different formulations in the stomach compartment over a period of three hours with BCG survival in the stomach effluent assessed after each hour (Fig 5A). Although the small sample sizes prevent robust statistical assessment, when expressed as a cumulative percentage of the intake, the amount of BCG recovered from the stomach appeared to be lower for BCG with PT bait compared with HPO-PT bait or when bait wasn’t present, consistent with the data shown in Fig 4. In both studies, we assessed BCG survival from the stomach through to the jejunum (Fig 5B). Most of the surviving BCG bacteria left the jejunum compartment during the first hour. In the second hour a smaller percentage of living BCG left the jejunum compartment. Only for the BCG without bait did the sample of the third hour contain additional living BCG bacteria. Recovery of BCG from the stomach was significantly greater when no bait was present (Fig 4B), but by the time of delivery to the jejunum there was no significant difference between the formulations in either study (2-way ANOVA). The difference seen in the stomach compartment could be the consequence of differences in the ability of the different formulations to buffer against acidity. In all experiments where it was examined, gastric pH reduced slowly during the first hour of the meal, more rapidly during the second hour, and then reduced more slowly for the final third hour (Fig 6). This profile was slower than the gastric pH profile observed *in vivo* using the Bravo device (Fig 1). There was no obvious difference in the pH profile between formulations, although during the second hour there was the suggestion that BCG without bait might buffer slightly better in a couple of the experimental runs.

Focusing on the stomach to jejunum, survival of BCG also appeared greater when bait was absent (Fig 4A and 4B). However, these differences were not statistically significant. There was also no significant difference in BCG survival in HPO-PT bait compared with CB-PT bait (Fig 4A) or PT bait (Fig 4B). CB-PT and PT were not compared within the same study.

The HPO-PT and CB-PT baits (two pieces were used for every TIM experiment) were 50–60% lipid by weight. Especially the HPO in bait had to be melted before it could be administered into the TIM stomach. During the runs with this bait the fat in the stomach was emptied into the duodenum compartment, but after the run had been completed it became clear that fat had accumulated at the side of the stomach and had become more solid again. The fat was removed from the stomach as much as possible after the run, and the residues were also plated to see if living BCG was present there. The quantity of BCG remaining in the stomach three hours after feeding with BCG-HPO-PT ranged from 4.1 x 10^2^ to 3.9 x 10^3^ CFU across both studies. The bait containing CB melted easier because of the lower melting point of the fat. Therefore less fat was expected to be left behind in the stomach compartment, but in one of the runs quite some fat accumulated right under the pyloric sphincter just above the duodenum compartment. This fat was removed after the run and added to the residue which was plated after the run. No BCG was recovered from this residue. Similarly, stomach residue after consumption of BCG with PT bait contained no viable bacteria. The highest numbers of living BCG were found in the stomach residues three hours after feeding without bait across both studies (2.2 x 10^4^ to 1.5 x 10^5^ CFU). However, by five hours no BCG was recovered from the stomach residues in seven of eight experiments. The exception was the recovery of 150 CFU BCG after five hours in the stomach without bait. Survival of BCG in the residues of the small intestine varied between formulations and between experiments and ranged from 2.2 x 10^3^ to 1.5 x 10^4^ CFU across both studies; the highest counts again being obtained where there was no bait.

## Discussion

We first performed simple *in vitro* experiments to ascertain whether simulated GIT conditions would cause a significant reduction in BCG viability and consequently could have a detrimental effect on the protective efficacy of an oral BCG vaccine. We found that the majority of BCG was killed following incubation with solutions intended to mimic the conditions of the stomach and intestine (simulated gastric and intestinal fluids, respectively). However, as these conditions were derived from experience with humans and dogs, we conducted remote-sensing experiments using a Bravo pH Monitoring System (Medtronic Ltd) to obtain more accurate physiological GIT data for the European badger. These experiments revealed that the gastric pH of captive badgers was around 2.0 for approximately 180 min in two badgers but only for about 40 min in another badger, where the pH rose to 6 and then fell again. Total gastric retention time was on average 480 min, but with a wide range (270–720 min). The pH of the small-intestinal tract was gradually increasing from 6.0 and 8.0 during transit through the duodenum, jejunum and ileum, with potentially long retention times (>10 hrs). These results are similar to the data obtained from Beagle dogs using the Bravo System [23, 24]. The gastric pH of the badger was at the higher end of the range found for fed or fasted Beagles and the gastric retention time was between that of fasted and fed dogs; being 250 min and 679 min, respectively. The pH and retention time of the canine intestinal tract was not the focus of study in the paper of Sagawa *et al*., but can be derived from figures therein [24]: pH of between 7 and 9; total (stomach + intestines) retention time up to 20 h. These data are very similar to those derived by us from badgers. We do not know the impact of general anaesthesia on gastric motility in the badger. We use a combination of 10 mg/kg of ketamine, 100 μg/kg of medetomidine and 100 μg/kg of butorphanol for anaesthesia. In a study with dogs, injection of up to 30mg/kg of ketamine did not significantly affect gastric emptying nor GIT motility [34].

We used the Bravo badger data to parameterise the TIM-1 system so we could make reproducible assessment of the viability of BCG vaccine as it passed through the stomach and small intestine sections of the model and what influence bait (type and presence) had on BCG survival. The precise conditions of the badger gut could not be replicated in terms of enzymatic secretions. Instead, we used the reagents typical of the canine configuration of the TIM-1 system [29] on the basis that the natural diet of dogs and badgers is not dissimilar; both being evolutionary omnivores. Microbial contamination of the model, after as much disinfection as possible, was not a problem and BCG bacteria could be recovered from the samples.

The gastric environment was most detrimental to BCG survival. As BCG is unable to maintain intracellular pH homeostasis under conditions of particularly low pH [35], the acidic conditions of the stomach are the most plausible explanation for our findings. However, the finding that survival was reduced in the presence of bait was surprising and runs contrary to previous data that suggested that the ingestion of live BCG vaccine with lipid might protect against inactivation in the stomach [36], although different a different composition of lipid was used in that study. The detrimental impact HPO-PT and PT baits had on BCG survival in the stomach was not related to pH as there was no obvious impact of bait present at the same time as BCG on the pH kinetics in the stomach. Although our data indicate that BCG without bait might buffer slightly better in a couple of experiments, the differences were small and we consider unlikely to account for the better survival of BCG when not in bait. One possible explanation for our findings is that free fatty acids released by digestion within the stomach of TIM-1 might have had an antimicrobial effect, especially longer-chain fatty acids (C12 and above) that have been shown to be toxic to BCG [37]. Coconut oil is rich in C12 and C14 fatty acids, whilst peanut oil is richer in C18 fatty acids, in particular [38]. However, other lipid matrices used to suspend BCG for oral vaccination have proved successful, despite over 78% of the composition of one of those matrices comprising lipids of C12-18 chain length [36]. A refinement in further studies would be to mix the BCG suspension with the ‘chewed’ bait before introducing into the stomach compartment. This difference might influence the release and survival of the BCG bacteria during GI transit and give different results.

Further reduction in BCG survival was encountered in the intestinal region of TIM-1 but not to the same extent as the stomach. Previous *in vitro* work has indicated a role for both gastric and duodenal fluids in causing significant BCG death [21], something we also observed in this study. As there is a very large Peyer’s Patch (lymphoid tissue responsible for uptake and presentation of BCG from the gut) about 33cm long at the distal end of the small intestine of badgers [39], the extent of survival of BCG in the jejunal effluent is the most important read-out from the TIM-1 badger model. The log_10_ reduction in BCG survival from ingestion to exit from the jejunum varied from 1.9 to 2.7 and was not significantly influenced by the presence or absence of bait, nor the type of bait used (i.e. the proportion and type of fat present). This could have important implications for the development of a cost-effective oral vaccine for badgers. We do not currently know the minimum efficacious dose of oral BCG for use in badgers, but published studies and unpublished studies by us suggest it may lie between 5 x 10^7^ and 10^8^ CFU. Our data suggest that around 2 logs of ingested BCG could be killed before delivery to the gastric-associated lymphoid tissue. As we currently estimate that BCG is the larger proportion of the cost of the vaccine bait (the bait components used in this study are relatively cheap), there are economic incentives for trying to reduce the effective dose of BCG needed to vaccinate badgers. Our findings imply this could be achieved if we were better able to protect BCG from the harsh environment of the stomach and duodenum.

A limitation of the experimental set up was the propensity for fats contained in the baits, HPO especially, to solidify and adhere to internal surfaces of the TIM-1 system. The presence of fatty deposits within the samples will also have reduced our efficiency in recovering BCG, especially if the naturally hydrophobic bacteria have a propensity to partition with the lipid. Retention of BCG in residues attached to internal surfaces of the TIM-1 system during our experiments will increase the apparent reduction in BCG survival, however, the number of bacteria retained in the residues of the stomach and intestinal compartments only represented a range from 0.7% (BCG-HPO-PT) to 6% (BCG-no bait) of the total CFU recovered from the system after five hours so have little overall impact on our findings. Furthermore, the high reproducibility between the two studies, for BCG with HPO-PT and BCG without bait, also suggests these practical limitations did not hamper us from achieving consistent and meaningful results.

Given that uptake of BCG from the intestine appears to be relatively inefficient [21], efforts to improve the survival of BCG within the GIT could result in formulations that are effective at lower doses. The observation that BCG given orally can be recovered from the cervical lymph nodes of mice, guinea pigs and humans [40–42] implies that the oropharynx is an additional portal of entry for BCG to the host mucosal-associated lymphoid tissue. Given the relative inefficiency of oral BCG vaccination that relies upon uptake from the intestine, more effort to understand the uptake of BCG from the oropharyngeal region could prove fruitful. Our studies are timely, given the renewed interest in the oral delivery of BCG to humans [43]. We conclude that the TIM-1 system provides a valuable, non-animal model with which to evaluate and optimise formulations to maximise BCG survival in the GIT.

## Supporting information

S1 Table Composition of simulated gastric fluid. (DOCX)

S2 Table Composition of simulated intestinal fluid. (DOCX)

S1 Fig Photograph of the TIM-1 system alongside a schematic representation of TIM-1. A. stomach compartment; B. pyloric sphincter; C. duodenum compartment; D. peristaltic valve; E. jejunum compartment; F. peristaltic valve; G. ileum compartment; H. ileo-caecal sphincter; I. stomach secretion; J. duodenum secretion; K. jejunum/ileum secretion; L. pre-filter; M. semi-permeable membrane; N. water absorption; P. pH electrodes; Q. level sensors; R. temperature sensor; S. pressure sensor.
(TIF)

S1 Dataset Raw data used to generate Fig 2. (CSV)

S2 Dataset Raw data used to generate Fig 4A. (CSV)

S3 Dataset Raw data used to generate Fig 4B. (CSV)
