# Supplementary material for: Survival of Mycobacterium bovis BCG oral vaccine during transit through a dynamic in vitro model simulating the upper gastrointestinal tract of badgers
Source: PLoS One. 2019 Apr 19;14(4):e0214859. doi: 10.1371/journal.pone.0214859 (PMC6474584; doi:10.1371/journal.pone.0214859)
Supplement: S1 Table — (DOCX) [file pone.0214859.s001.docx]

| Sodium chloride | 0.03 M | Sigma. Catalogue number: S6191;  Biotechnology Performance Certified ≥ 99.5%. |
| --- | --- | --- |
| Pepsin | 0.32% (w/v) | Sigma. Catalogue Number: P7000;  800-2,500 U mg^-1^ protein. |
| Gastric Lipase  (canine)- | 115 μg ml^-1^ | Meristem Therapeutics*.  Freeze dried product, 400 U mg^-1^ protein. |
| Hydrochloric acid | Used to adjust pH | Sigma. Catalogue Number: H1758;  Molecular Biology Grade, 36.5-38.0%. |
| pH | 5.0 & 1.2 | Hold pH at 5 for first 135 min and then at 1.2 for 165 min. |
| Temperature | 37° C |  |

*Meristem Therapeutics, Clermont-Ferrand, France.
