# Supplementary material for: Survival of Mycobacterium bovis BCG oral vaccine during transit through a dynamic in vitro model simulating the upper gastrointestinal tract of badgers
Source: PLoS One. 2019 Apr 19;14(4):e0214859. doi: 10.1371/journal.pone.0214859 (PMC6474584; doi:10.1371/journal.pone.0214859)
Supplement: S2 Table — (DOCX) [file pone.0214859.s002.docx]

| Monobasic potassium phosphate (KH_2_PO_4_): | 0.05 M | Sigma. Catalogue Number: P5655;  powder ≥ 99.0%, cell culture tested. |
| --- | --- | --- |
| Pancreatine | 10 mg ml^-1^ | Sigma. Catalogue Number: P1500. |
| Colipase | 20 μg l^-1^ | Sigma. Catalogue Number: C3028;  from porcine pancreas, lyophilised. |
| Pancreatic lipase | 300 μg l^-1^ | Sigma. Catalogue Number: L3126;  from porcine pancreas, contains amylase and protease activity. |
| Sodium cholate | 2.7 mg ml^-1^ | Sigma. Catalogue Number: S9875; contains the sodium salts of taurocholic, glycocholic, deoxycholic, and cholic acids. |
| Sodium hydroxide | See below | Sigma. Catalogue Number: S8045; Sigma Ultra, ≥ 98%, pellets (anhydrous). |
| pH | 7.5 | Adjusted with sodium hydroxide. |
| Temperature | 37° C |  |
