# Supplementary figures and images for: Survival of Mycobacterium bovis BCG oral vaccine during transit through a dynamic in vitro model simulating the upper gastrointestinal tract of badgers
Source: PLoS One. 2019 Apr 19;14(4):e0214859. doi: 10.1371/journal.pone.0214859 (PMC6474584; doi:10.1371/journal.pone.0214859)

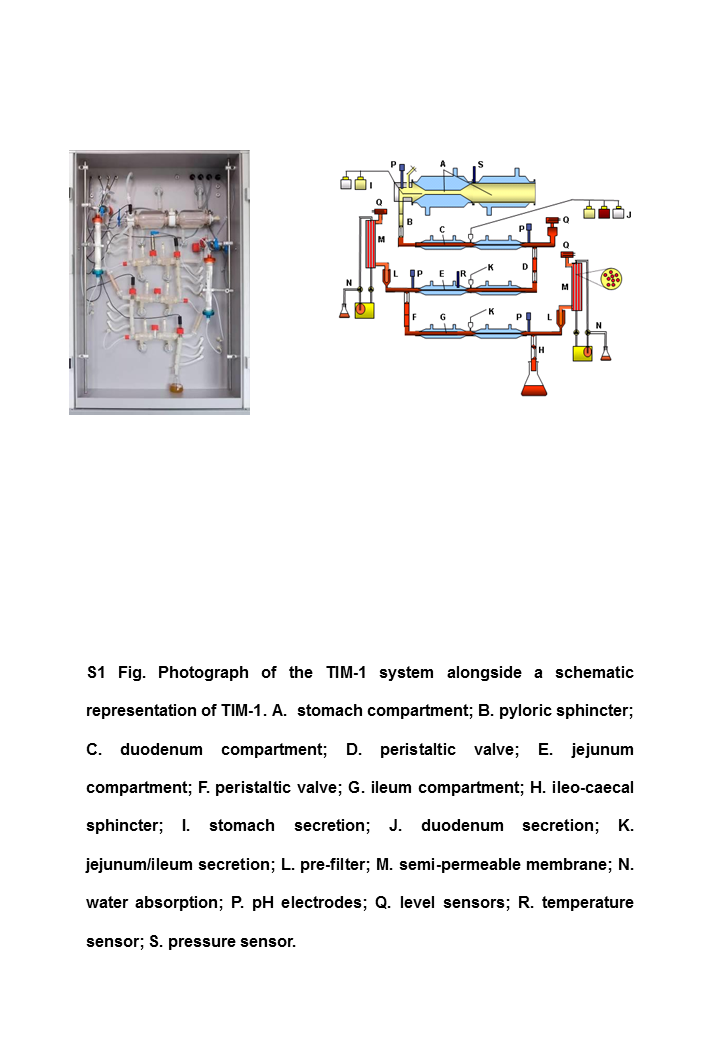

Supplement: S1 Fig — A. stomach compartment; B. pyloric sphincter; C. duodenum compartment; D. peristaltic valve; E. jejunum compartment; F. peristaltic valve; G. ileum compartment; H. ileo-caecal sphincter; I. stomach secretion; J. duodenum secretion; K. jejunum/ileum secretion; L. pre-filter; M. semi-permeable membrane; N. water absorption; P. pH electrodes; Q. level sensors; R. temperature sensor; S. pressure sensor. (TIF) [file pone.0214859.s003.TIF]
